# Underwater Crawling Robot With Hydraulic Soft Actuators

**DOI:** 10.3389/frobt.2021.688697

**Published:** 2021-08-26

**Authors:** Qinlin Tan, Yishan Chen, Jianhui Liu, Kehan Zou, Juan Yi, Sicong Liu, Zheng Wang

**Affiliations:** ^1^ Department of Mechanical and Energy Engineering, Southern University of Science and Technology, Shenzhen, China; ^2^ Guangdong Provincial Key Laboratory of Human Augmentation and Rehabilitation Robotics in Universities, Southern University of Science and Technology, Shenzhen, China; ^3^ Shenzhen Key Laboratory of Biomimetic Robotics and Intelligent Systems, Department of Mechanical and Energy Engineering, Southern University of Science and Technology, Shenzhen, China

**Keywords:** underwater robot, soft robotics, untethered robot, crawling robot, hydraulic actuation

## Abstract

Benthic operation plays a vital role in underwater applications, where crawling robots have advantages compared with turbine-based underwater vehicles, in locomotion accuracy, actuation efficiency, current resistance, and in carrying more payloads. On the other hand, soft robots are quickly trending in underwater robotic design, with their naturally sealed body structure and intrinsic compliance both desirable for the highly unstructured and corrosive underwater environment. However, the limitations resulting directly from the inherent compliance, in structural rigidity, actuation precision, and limited force exertion capability, have also restricted soft robots in underwater applications. To date soft robots are adopted mainly as grippers and manipulators for atraumatic sampling, rather than as locomotion platforms. In this work, we present a soft-robotic approach to designing underwater crawling robots, with three main innovations: 1) using rigid structural components to strategically reinforce the otherwise omni-directionally flexible soft actuators, drastically increasing their loading capability and actuation precision; 2) proposing a rigid–soft hybrid multi-joint leg design, with quasi-linear motion range and force exertion, while maintaining excellent passive impact compliance by exploiting the inherent flexibility of soft actuators; 3) developing a novel valve-free hydraulic actuation system with peristaltic pumps, achieving a compact, lightweight, and untethered underwater crawling robot prototype with a 5:1 payload-to-weight ratio and multi-gait capability. The prototype was tested for design verification and showcasing the advantages of the proposed hybrid mechanism and actuation approach.

## Introduction

Soft robotics is a top-trending area, inspiring paradigm changes in robotic design and actuation, with soft-material bodies of inherent compliance being used both as structural components and as actuators (Rus and Tolley, 2015; Wang et al., 2015; Liu et al., 2021). There have been many notable attempts in developing underwater soft robots, but mostly focused on the manipulation and sampling end of operations, ranging from underwater soft actuators (Ku et al., 2009; Yim et al., 2007), wrist joint (Kurumaya et al., 2018), manipulator arms (Phillips et al., 2018; Gong et al., 2019; Shen et al., 2020), and underwater exploration platforms (Patterson et al., 2020). These underwater soft robot designs showcased their advantages for the underwater environment: soft manipulators or grippers composed of simple structural design and modular variants could achieve both a high degree of dexterity and flexibility (Phillips et al., 2018; Gong et al., 2019; Shen et al., 2020). In particular, the soft actuators’ natural waterproofness, superior environmental adaptability, and flexibility make them ideal for interacting with various marine creatures within the pressurized and corrosive harsh underwater environment (Calisti et al., 2011; Cianchetti et al., 2011; Arienti et al., 2013; Stilli et al., 2014; Stuart et al., 2014; Shen et al., 2021). Soft actuators are the core to the unique soft robotic features. With their bodies entirely composed of thin-shell soft materials, they followed a completely distinctive approach to robotic actuators by offering high force output compared with their own weight at a fraction of the hydraulic actuation fluids being supplied. This often resulted in drastic size/weight advantages, in addition to the inherent compliance deduced from the soft structures, when compared with hydraulic cylinders and servo motors commonly used in composing underwater crawling robots. Also, the use of hydraulic cylinders requires more complicated control, high cost, and high noise (Ku et al., 2009).

However, despite their success as manipulators and sampling grippers, existing underwater soft robotic limbs still require conventional robotic vehicles as mounting platforms (Phillips et al., 2018; Gong et al., 2019; Shen et al., 2020), while existing soft underwater vehicles or robotic platforms are highly scarce, with highly limited payload or locomotion capabilities (Ishida et al., 2019; Patterson et al., 2020), insufficient for carrying soft manipulators. The key bottle neck of soft robots for payload-and-precision demanding tasks such as marine benthic crawling lies in the soft actuators. With unregulated, omni-directional passive compliance, soft actuators by default could not achieve directional actuation without being soft and flexible in other directions, therefore significantly hindering their levels of force payload and precision (Zhou et al., 2018). Using rigid reinforcements on soft actuators could significantly constrain their compliance and achieve directionality during operation, the approach has been repeatedly proven using pneumatic soft robots as wearable devices and industrial grippers (Zhou et al., 2018; Liu et al., 2020; Su et al., 2020), achieving dozens of times payload increase without sacrificing lightweightness. However, for underwater applications very little work was reported to date.

In this work, we explore a fusing design of soft actuators augmented into rigid structural components strategically, to form rigid–soft hybrid legs, and develop matching actuation systems and controls, toward multi-gait, high-payload crawling robots in contrast to the existing underwater soft robots. The core spirit of this work is to harness the excessive compliance toward the desired direction and amount using proper constraints and actuation. The main contributions of this work are as follows:

1) Proposed a novel underwater robot leg design using hydraulic hybrid-soft actuator (H^2^SA) joints. In contrast to the unconstrained soft actuator joints or the individually reinforced soft actuators used in state-of-the-art works, this approach took the structural components of the leg as the main frame of reinforcement, and augmented the soft actuators into the leg strategically to achieve the desired kinematics, range of motion, and the payload capability, while maintaining sufficient levels of passive compliance for impact safety and adaptation in unstructured environments. Analysis of a design strategy was presented, together with a working design used in prototype development.
2) Matching with the proposed leg design, a novel compact and multi-channel direct pump pressurization (DPD) actuation system was developed. To achieve large range of motion, both positive and negative pressurization were enabled for each soft actuator to drive it to the full deformation range. However, aiming for compactness and light weight, in this work we proposed to use peristaltic pumps to drive the soft actuators directly, rather than using the conventional pump-valve approach. By using minimum actuation components (one pump per each independent DOF), we could reduce system size/weight, as well as simplify the actuation procedure.
3) An untethered underwater crawling (U^2^C) robot was built with H^2^SA legs and DPD actuation, to showcase the unique features and characteristics of the proposed design approaches. The U^2^C robot was untethered with self-containing actuators, electronics, and battery. It could achieve multiple gain patterns with the six H^2^SA legs consisting of 18 joints and DPD actuation. By introducing the H^2^SA design, the force-enhanced H^2^SA legs achieved drastically higher payloads at lighter weights, achieving 15 times maximum payload to its own weight, significantly improving over state-of-the-art soft underwater robots. At the same time, the legs remained compliant to impacts to ensure safe benthic explorations.

This article is organized as follows: The concept of H^2^SA leg and joint is introduced in *Design Concept of the Crawling Robot With H* ^*2*^ *SA Legs and Soft Joints*. *Modeling* presents the design and modeling details of the H^2^SA joint and H^2^SA leg. *Fabrication and experiment validation* presents the fabrication of the U^2^C robot, together with a series of verification experiments and demonstrations with static analysis. Conclusions and future work are given in *Conclusion and future work*.

## Design Concept of the Crawling Robot With H^2^SA Legs and Soft Joints

The core spirit of the proposed H^2^SA approach is to augment soft actuator compliance with rigid constraints, so that substantially improved performance indices (such as rigidity, payload, precision, etc.) could be achieved while maintaining the desirable features such as passive adaptation and compliance. To achieve this goal, we sought for a very simple, conventional, and well-studied soft bellows (Wang and Wang, 2020) as the choice of soft actuators, and reinforced them strategically using leg structural components, and then assessed the resulting kinematic performances and features.

### Spider-Inspired Leg Kinematic Design

**FIGURE 1:**
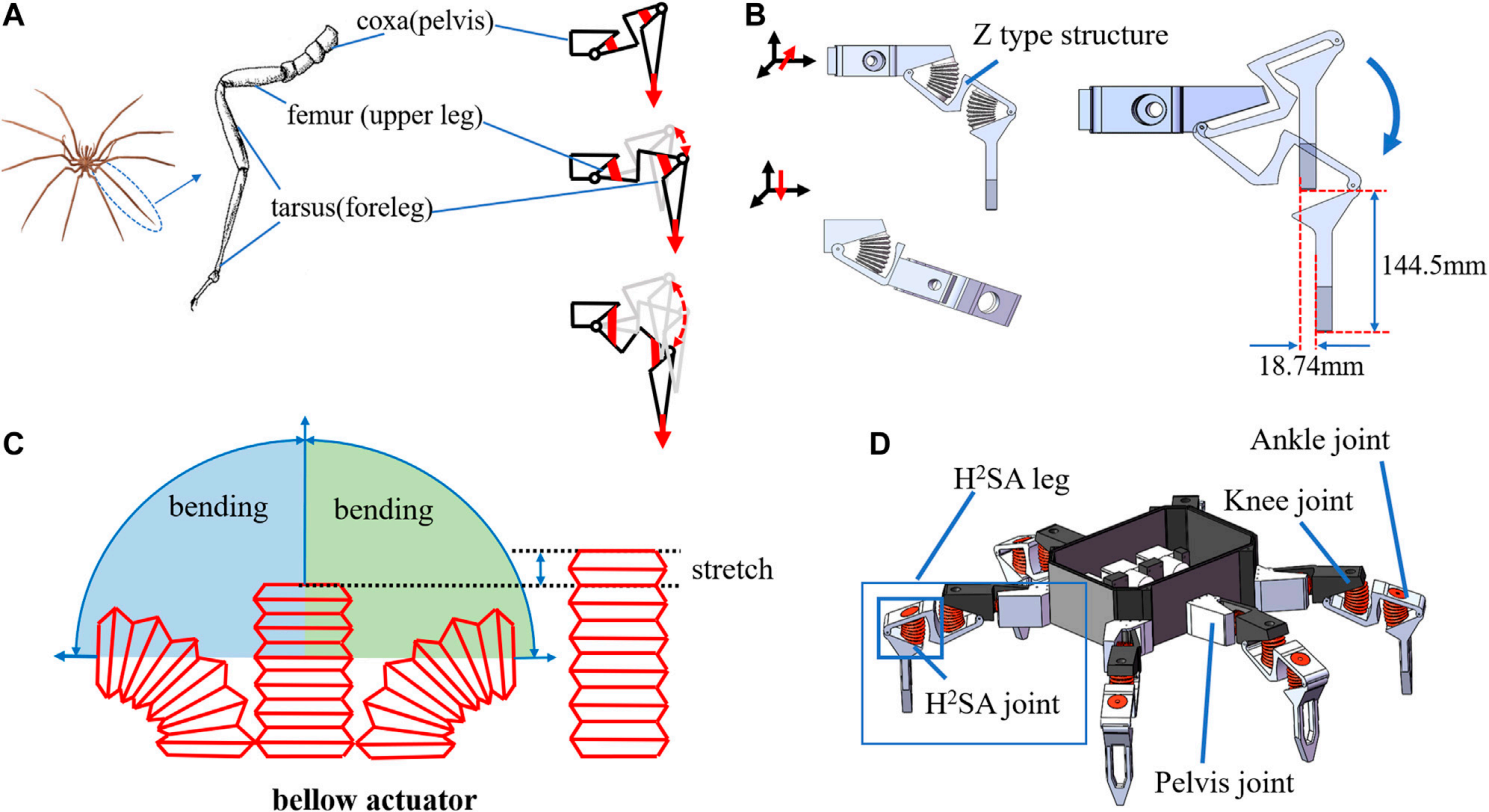
Concept of soft underwater crawling robot: **(A)** illustration of one-leg structure and gait; **(B)** single-leg 3D model; **(C)** movement characteristics of bellows actuator; **(D)** conceptual design of a proposed soft underwater crawling robot.

For the kinematic design of a poly-limb underwater crawling robot, spiders are natural sources of inspiration, both for their iconic tetra- to octo-pod body structure and gaits, and for their wide range of habitats covering both on land and in water (Wang and Wang, 2020; Ballesteros et al., 2021). The hexapod/octopod gaits of spiders with benthic-facing tarsus/metatarsus segments (Figure 1A) could provide excellent traction on the sediment-filled sea floor (Zentner, 2013), but soft actuators with continuum flexible bodies could not reproduce such gaits, only sweeping across a spherical surface (Patterson et al., 2020) rather than following a linear path (Figure 1B). The continuum soft body of soft actuators also makes them inherently prone to external force exertions, causing undesired deformations on non-actuating directions, as well as hindering the force output capability.

Therefore, it is essential to underpin the kinematics of the leg with a rigid, hinged leg structure, and then augment it with soft muscles for actuating the joints. To this end, we took inspiration from the sea spider, and proposed a leg kinematic design with the following main segments (Figure 1B):

1) The foreleg, corresponding to the tarsus/metatarsus of the spider, touching the ground surface during locomotion;
2) The upper leg, corresponding to the femur of the spider, enabling the foreleg to extend away from the torso for better stability during walking;
3) The pelvis, corresponding to the coxa of the spider, for lateral swinging of the leg.
4) Connecting the three sections are the three soft-robotic joints, one for the pelvis, one for the knee, and one for the ankle.

Although this kinematic structure is common among motor-driven hexapod/octopod robots, here the main challenge lies in the augmentation of soft actuators with the rigid structural mechanisms toward desirable motion specifications. Therefore, a number of design innovations were made to incorporate the soft actuators with their unique characteristics.

### Augmenting the H^2^SA With Soft Actuators

There are abundant choices of soft actuators to drive a revolute joint. In principle the presented concept has no restrictions on the choice of actuators, as long as they could fit the dimensions and generate elongating deformation when actuated.

Here for the sake of generality, we use one of the simplest and legacy types of soft actuators with bellows chamber as the actuator of choice. As thoroughly studied previously, such actuators have excellent range of motion, repeatability, and large force output, while also being known for suffering from poor lateral stiffness and are omni-directionally flexible (Zhou et al., 2020). The bellows actuator will expand and contract along the axis when it is not restrained, and output the main force in this direction. When the two ends are restrained, it can shrink in the direction of the arc formed after restraint (Figure 1C). The leg design innovations to augment soft muscles are as follows:

1) Orthogonal alignment of pelvis and knee joints, to decouple the compliance along the two perpendicular directions. By enabling the revolution of the two joints on the two primary motion directions and constraining all other directions, each joint is fully constrained and free from any unwanted flexibility other than the actuated direction, but the overall leg is still compliant both horizontally (the remaining compliance of the pelvis joint) and vertically (from the knee);
2) Conjugate planar alignment of the knee and ankle joints, to couple the two joints deeply, to achieve a quasi-linear motion path along the vertical axis. Although each soft actuator is nonlinear and passively compliant, their nonlinear and compliant characteristics are repeatable with low inter-sample variation. Therefore, by conjugating two actuators, we could counter each other for their nonlinearity and compliance, and yielding a linear motion on the foreleg;
3) A Z-shaped upper leg segment with an inverted middle section, to house two soft bellows actuators with larger diameters while maintaining a compact overall length. Since the force output of soft bellows actuators is directly corelated with their diameter, it is desirable to maximize the actuator diameter within the available space. Therefore, the middle section of the Z-shaped upper leg was inverted, such that the length of the upper leg segment could be fully utilized by the two actuators without interfering each other during motion.

The resulting H^2^SA leg design, as shown in Figure 1B, was composed around a Z-shaped planar two-bar linkage, where two independently actuated opposing soft actuators were mounted in the ridges of the Z-shaped piece. The resulting two-bar linkage could generate a quasi-straight trajectory along the vertical axis. In particular, the leg configuration shown in Figure 1B could achieve a vertical motion range of 144.5 mm, while the maximum lateral displacement was 18.74 mm, which is 12.9% of the vertical motion range. The six legs are shown in Figure 1D to form a hexapod underwater crawling robot platform. Further modeling and analysis on how to strategically locate the soft muscles inside the leg will be presented in *Modeling*.

## Modeling

To better understand the performance of H^2^SA leg and joint, this section investigated the relations of characteristics and design parameters. The modeling of H^2^SA joint involves motion range, load capacity, and variable compliance based on design parameters. Based on the joint models, we investigated the movements and load of H^2^SA leg. The proposed modeling work could provide guidance for the joint, leg, and robotic system design.

### Modeling of H^2^SA Joint

**FIGURE 2:**
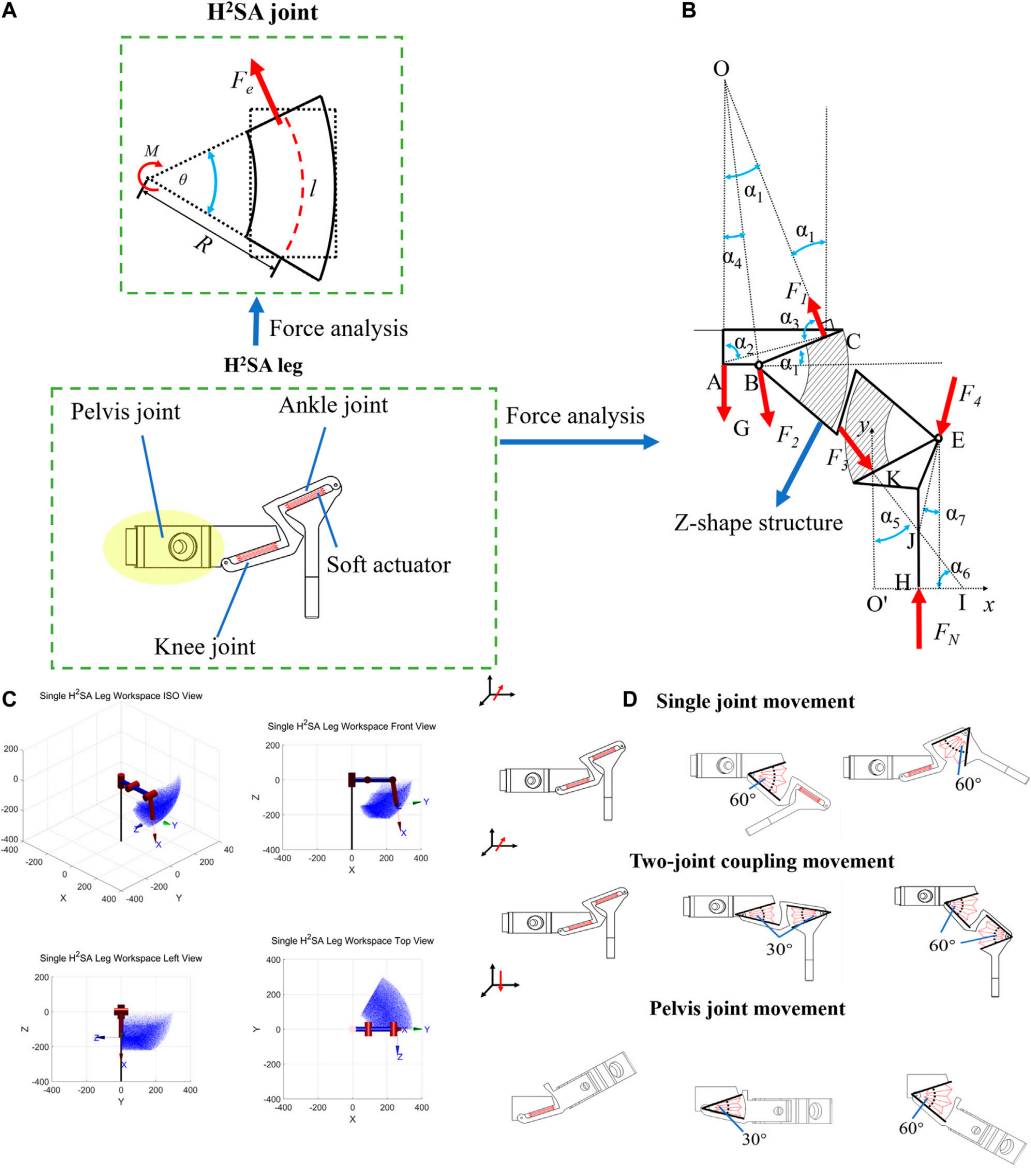
Modeling of H2SA joint and leg: **(A)** force analysis of a single joint; **(B)** force analysis of two joints on a single leg; **(C)** working space simulation; d) H^2^SA leg model and movement.

As the basic actuation units of robot, the key for H^2^SA joint design is to reveal the relations of output force, motion range, and design parameters. A H^2^SA joint consists of a soft actuation bellows and a rigid joint. By constraining the linear soft bellows into a rotational joint, rotational actuation could be achieved characterized by a torque M and angle θ that could be tuned by design parameters, including the radial distance from joint center to soft actuator bellows R, axial length of bellows l and water pressure P, as shown in Figure 2A. The relations of M, θ, and $F_{e}$ could be presented as

$$M=F_{e}R\;$$ (1)

where $F_{e}$ is the generated force of soft bellows with a direction perpendicular to the bellows cap, satisfying

$$F_{e}=k\left(l-l_{0}\right)+PS$$ (2)

where k is the stiffness mainly depending on the structure and materials of bellows. $l_{0}$ is the original axial length of bellows at the natural state. S is the cross-sectional area of bellows cap (Wang and Wang, 2020). Substituting (2) into (1), we have

$$M=\left(k\left(l-l_{0}\right)+PS\right)R\;$$ (3)

The geometrical relations between l, θ, and R could be analyzed as

$$\theta =\frac{180{}^{\circ}l}{\pi R}$$ (4)

Analyzing (3) and (4), larger torque M could be obtained from a larger R, while opposite tendency is shown between smaller angle θ and R. Therefore, to achieve a desirable larger torque and an angle, a suitable R should be calculated after considering the requirements of robot.

### Modeling of H^2^SA Leg

The compliance and load capacity of robotic system mainly depends on the robot’s gait where static models of leg are investigated. In this part, the H^2^SA leg is composed of three H^2^SA joints. The knee joint and the ankle joint are connected by a Z-shaped structure that allows the legs to keep the toes straight down, while the knee and ankle joints move in the same state. The force analysis of a single H^2^SA leg based on the modeling work of joint, as shown in Figure 2B, where the bold black line represents the rigid structure, the circle represents the rotation pair, the shadow represents the soft actuator, and the red arrow represents the force. The relationship of the angles and lines can be determined according to the trigonometric relationship:

$$OA=AC\frac{\sin\alpha_{3}}{\sin\alpha_{1}}\mathrm{, tan}\alpha_{4}=\frac{AB}{OA}$$ (5)

where $\alpha_{1}\;$is the angle between G and $F_{1}$, $\alpha_{4}$ is the angle between G and $F_{2}$, and $\alpha_{3}$ is the angle of ∠OCA,

Considering the force balance:

$$G+F_{2}\cos\alpha_{4}=F_{1}\cos\alpha_{1}$$ (6)

$$F_{2}\sin\alpha_{4}=F_{1}\sin\alpha_{1}$$ (7)

Substituting the parameters $\alpha_{1}=17.14{}^{\circ}$, $\alpha_{2}=78.52{}^{\circ}$, $\alpha_{3}=82.3{}^{\circ}$, AC=125.83mm, and AB=63.19mm into the equations, the relation of $F_{1}$ and G is:

$$G=-0.994F_{1}$$ (8)

The component EHK is similar to the above force analysis of the component ABC. J is the intersection point of the supporting force F_N_ and the force F_3_ applied by the component BE to the component EHK. F_4_ is the force applied to the component EHK by the component BE, pointing to J. Further analysis can be carried on by setting up the Cartesian coordinate system, whose origin is point $O^{'}$ , while its *y* axis passes vertically through point K and the x axis passes through point H horizontally. Point I is the intersection point of the $F_{3}$ and x axis. So, the coordinates of each point are as follows: $H\left(x_{H},\;0\right)$, $I\left(x_{I},\;0\right)$, $E\left(x_{E},\;\;y_{E}\;\right)$, $J\left(x_{J},\;\;y_{J}\;\right)$. The structural parameters are known as:$\;x_{H}=6.9\;mm$, $y_{K}=119.05\;mm$, $x_{E}=32.37\;mm$, $y_{E}=147.36\;mm$, $\alpha_{5}=24.59{}^{\circ}$.

By the geometric relationship:

$$\alpha_{6}=90{}^{\circ}-\alpha_{5}$$ (9)

$$\frac{y_{E}}{\tan\alpha_{6}}=x_{J}$$ (10)

$$\frac{x_{I}-x_{H}}{x_{I}}=\frac{y_{J}}{y_{K}}$$ (11)

$$x_{H}=x_{J}$$ (12)

Considering the force balance:

$$F_{N}=F_{4}\cos\alpha_{7}+F_{3}\cos\alpha_{5}$$ (13)

$$F_{4}\sin\alpha_{7}=F_{3}\sin\alpha_{5}$$ (14)

The position of point J can be determined:

$$\tan\alpha_{7}=\frac{x_{E}-x_{J}}{y_{E}-y_{J}}$$ (15)

Substituting the parameters $x_{H}=6.9\;mm$, $y_{K}=119.05\;mm$, $x_{E}=32.37\;mm$, $y_{E}=147.36\;mm$, $\alpha_{5}=24.59{}^{\circ}$.

Then, the force of $F_{N}$ can be finally determined:

$$F_{N}=1.618F_{3}$$ (16)

Eq. 8 gives the relationship between the load of the knee joint and the leg. Eq. 16 gives the relationship between the load of the ankle joint and the leg.

A simulation on workspace of a single leg is also carried out, with the results shown in Figure 2C. The diagrams of each joint’s movements are shown in Figure 2D, each joint can move independently.

## Fabrication and Experiment Validation

The fabrication and experimental validation of the proposed robots are presented in this section. First, life span, motion range, and the passive compliance of H^2^SA joint were validated. Then, the output force response of a single leg to the hydraulic pressure was measured before validating the motion of single leg of the robot. Then the load capacity of the robot system was tested. Finally, we demonstrate that the crawling robot could achieve different gaits on the water bottom with sediments and obstacles.

### Fabrication of the H^2^SA Joint

**FIGURE 3:**
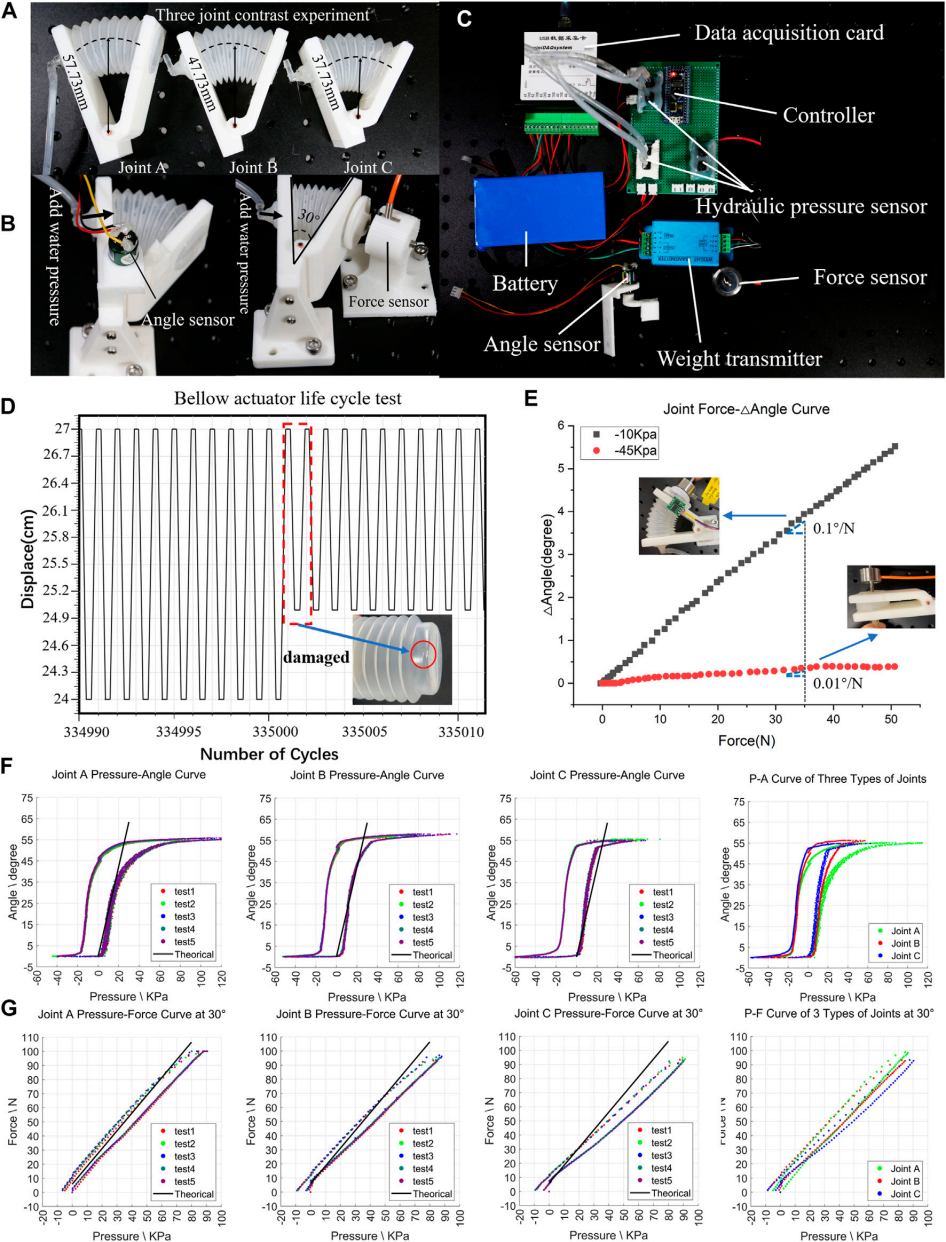
Test on H^2^SA joint. **(A)** Three kinds of joints: A joint, B joint, and C joint; **(B)** test angle/force and hydraulic relationship curve device for A joint, B joint, and C joint; **(C)** experimental test platform, including hydraulic pressure, force, and angle; **(D)** life cycle test result graph; **(E)** the relationship between force and △angle under different initial pressures; **(F)** comparison of the hydraulic pressure and angle curves of A joint, B joint, and C joint with theoretical values; **(G)** curve results and comparison of A joint, B joint, and C joint hydraulic pressure and force with theoretical values.

To compare the placement strategy of soft muscles into rigid joints, three different H^2^SA joint design prototypes (joint A, joint B, joint C, shown as Figure 3A) were fabricated to validate the joint models. In each design, the soft muscle had a different mounting distance from the joint rotation fulcrum. The H^2^SA joint is with a rigid–soft hybrid structure where the rigid skeleton is 3D printed with polylactic acid (PLA) materials. The soft bellows actuator is inject-molded with low-density polyethylene and glued to the rigid skeleton through ethyl vinyl acetate (EVA). Parameters of bellows are listed: $l_{0}=57mm$, $S=683.49\;mm^{2}$, and k=0.714N/mm , where k could be obtained from our previous study (Zhou et al., 2018). The radial displacements on setting Joint A, Joint B, and Joint C are$\;R_{A}=57.73\;mm$,$\;R_{B}=47.73\;mm$, and$\;R_{C}=37.73\;mm$, respectively.

### Tests on the H^2^SA Joint

In this section, first test the life span of the bellows actuator. In addition, to verify the relationship between the output force, range of motion, and water pressure, joint A, joint B, and joint C were tested by angle test, force test, and compliance test on the experimental equipment shown in Figures 3B,C.

To facilitate the various tests on the joint, a dedicated experimental platform was designed and built in this work (shown in Figure 3C), comprising of two hydraulic sensors, one of which has a range of 0–100 kPa and the other has a range of −100–0 kPa, one linear force sensor with range 0–100 N (DYMH-103, 10 kg, Daysensor), one linear infrared meter (Benewake, 0.1–12 m), and one angle sensor (PandAuto, 0–360°) mounted on the plate to provide real-time water pressure, pulling/pressing force, and joint angle recordings with data acquisition card (miniDAQsystem, eight channels, 0–5 V). In addition, a hydraulic system with valves and pumps was designed to provide quantitative water pressures. Data were collected and processed by micro controller (STM32).

First, the service life of the H^2^SA joint was accessed in a life-span test. The soft bellows actuator was mounted in a rigid joint frame in the experimental platform, and actuated repeatedly until some failure occurred. During the test, the actuator was set in free space and completed the periodic displacement range 30 mm/2s under −10 kPa pressure. The results are shown in Figure 3D, which illustrates the bellows actuators failed after the 335,000th cycle. At the 335,000th cycle, the cap of actuator was fractured mainly caused by the friction between actuator and mounted base, which could be further improved by optimizing the mechanical structures.

Further, to validate the unique compliance of proposed H^2^SA joint, a compliance test was conducted in isotonic state (Yi et al., 2018). In this test, we manually rotated the joint, while the inlet and outlet of the actuator were kept in closed. The force sensor and angle sensor recorded the external force with angle varying. The experimental results are shown in Figure 3E, where different gradients are depicted, validating the variable compliance of joint. Given the external force 35 N, the joint compliances are calculated as 0.1°/N and 0.01°/N in −10 kPa and −45 kPa.

Finally, for the angle and force tests (Figure 3B), three joints were set in free space and isometrically, respectively, to measure the relations between force, joint angles, and water pressure. A rotation angle test was conducted in free space by recording the angle change under increasing water pressure, while force test was conducted by reading force change with pressure varying in fixed joint angles. Each test was performed three times to ensure the repeatability of experimental results. The relations of angle and pressure, force and pressure with analysis, are shown in Figures 3F,G respectively, indicating the high repeatability. Comparing the experimental results of the three joints, it can be seen that larger R would result in smaller θ and larger $F_{e}\;$by giving the same pressure. This tendency is consistent with model (3) and model (4). Slight deviations could be observed between the experimental and modeled results, possibly caused by the nonlinearity of structural deployment of bellows. A short conclusion could be made that Joint B is a better case in our design for a larger angle and a larger force output. Therefore, Joint B was selected as the H^2^SA joint for the composition of the crawling robot prototype.

### Fabrication and Tests of the H^2^SA Leg

**FIGURE 4:**
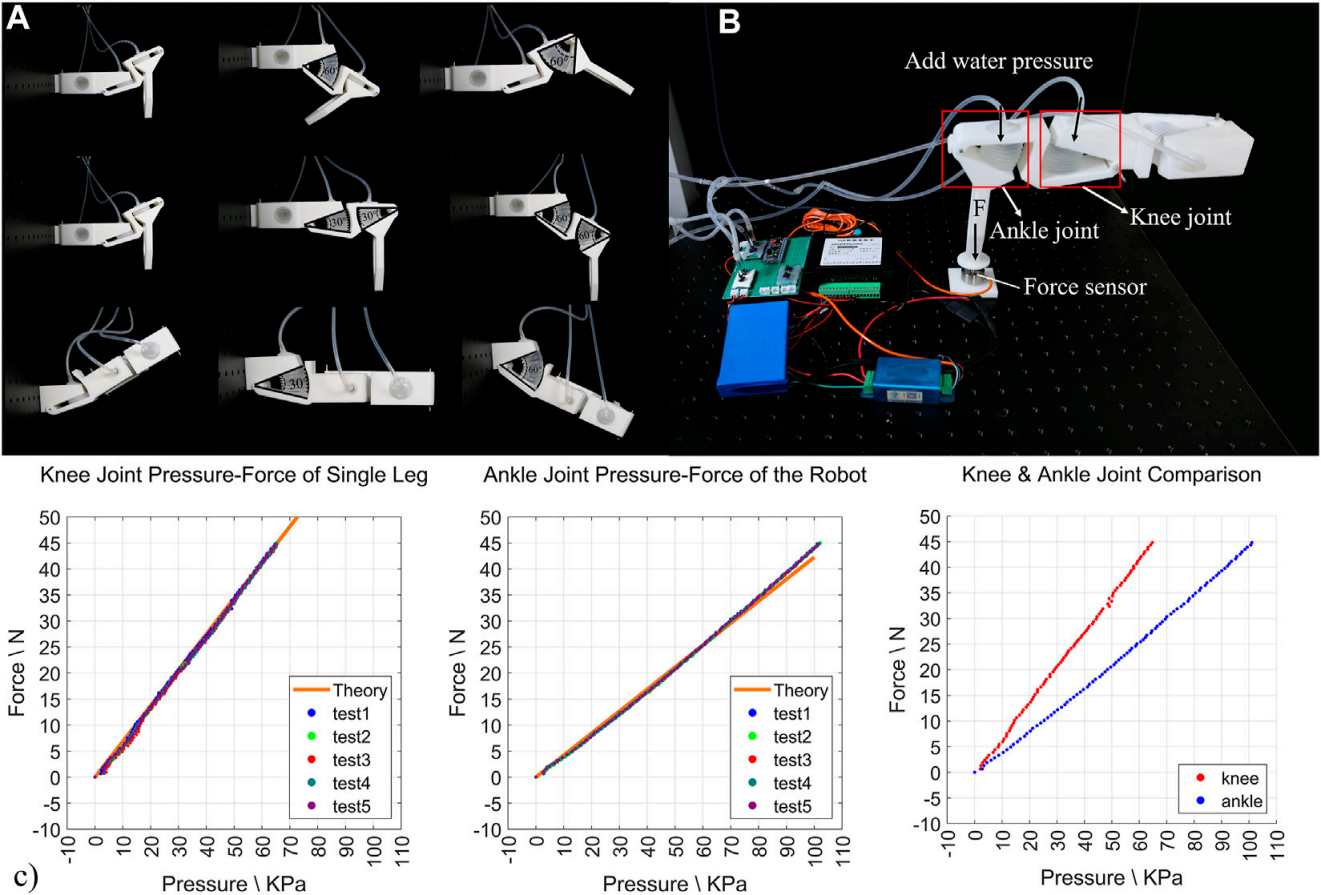
Test on H^2^SA leg. **(A)** The posture of the leg during the movement of each joint on the leg; **(B)** single-leg load test device; **(C)** the relationship between knee joint hydraulic pressure and ankle joint hydraulic pressure and leg load, and the comparison of the two joint curves.

A single H^2^SA leg consists of pelvis joint, knee joint, and ankle joint that use the same materials and dimensions as joint B. The knee joint and the ankle joint are connected by a Z-shaped rigid link, which ensures that the legs formed by the two joints have both compliance and load capacity when coupling motion (Figure 4A). Tests on the H^2^SA leg were conducted in a dedicated testing platform shown in Figure 4B. Tests on leg load capacity were conducted to validate Models (11) and (19). In this test, one end of the single leg was fixed on a vertical surface, and the other end was pressed against the ground through a force sensor. Two pressure sensors measure the internal pressure of two soft actuators (Joint knee and Joint ankle). Therefore, the relationship between the hydraulic pressure of the actuators and the output force of the end of the leg can be measured. In the test, we pressurized the actuator of Joint knee only and obtained the output force under different hydraulic pressures.

The test results are shown in Figure 4C where the experimental results were in good agreement (RMSE < 2.5%) with the theoretical calculations in both knee joint and ankle joint. Similarly, in the case of pressurizing the actuator of the Joint ankle only, the experiment data and analysis result are shown as the dots and solid line receptively in Figure 4C. The data are also very consistent, with RMSE less than 3%, indicating the correctness of force analysis and large load capacity.

### Design and Fabrication of U^2^C Robot

**FIGURE 5:**
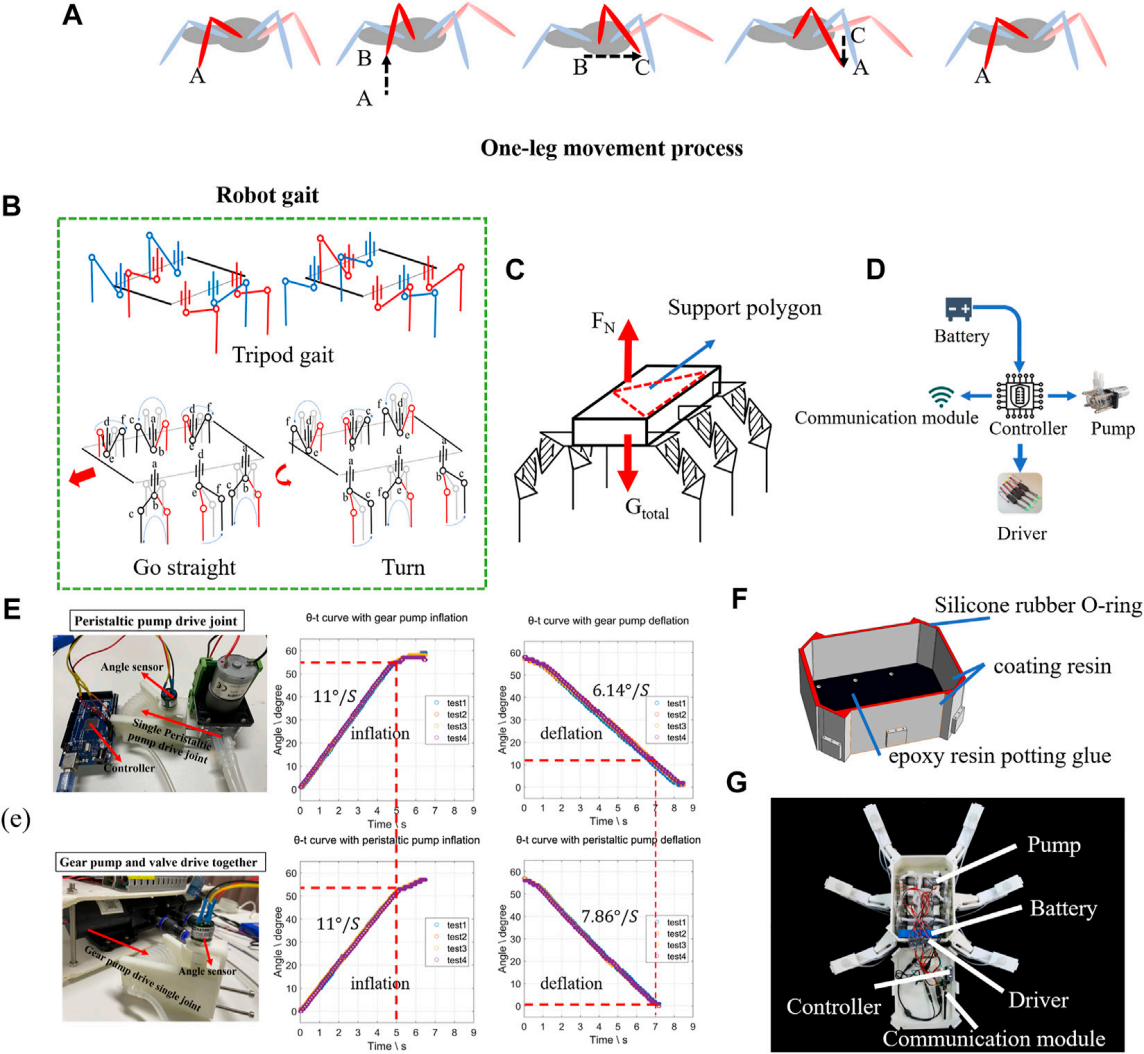
Design and fabrication underwater crawling robot. **(A)** Sea spider’s one-leg movement. **(B)** The alternating gait of a robot in motion. **(C)** Force analysis of a robot system; **(D)** a robot control system. **(E)** Response test of different pump valve systems. **(F)** Waterproofing of robots. **(G)** The underwater robot system.

Based on the previous design and validation test results, we finally compose the U^2^C robot with the selected leg design into a working prototype for further experimental validations on the proposed approach. The overall platform design inspiration still arose from the sea spider (the procedural detail of a spider taking one step is shown in Figure 5A). It is reliable to use alternate gait in footed robots (shown in Figure 5B) (Shepherd et al., 2011). To showcase the leg performance, rather than comparing gait patterns, for simplicity and without losing generality, the proposed robotic design consisted of six identical H^2^SA legs, mounted on the main body torso, where the six legs were grouped into pairs for alternate sequenced locomotion, for achieving both static stability and adaptability (Walas et al., 2013). During these movements, as shown in Figure 5C, the Center of Gravity (COG) usually falls into area of support polygon (Mishra, 2014). Analyzing the force distribution of the robot, the load capacity of the robot could be calculated

$$F_{N,total}=G_{total}=G+6\;G_{leg}$$ (17)

when the robot is standing with six legs.

$$\;F_{N,total}=G_{total}=G+3\;G_{leg}$$ (18)

when the robot is walking with three legs.

With the excellent force output capability of the soft actuator and the H^2^SA legs, the resulting U^2^C robot could carry all the necessary components required to sustain its motion, and therefore become truly untethered underwater. To achieve the untethered target, first the torso compartment needed to be fully waterproof, and the entire actuation and control system needed to be compact and lightweight enough to fit into the torso compartment, accordingly.

To achieve a compact and lightweight core actuation system, we chose to use direct pump drive (DPD), using peristaltic pumps to directly drive the joints, and completely remove any hydraulic valves from the hydraulic control loop. This could both avoid the small diaphragm flow restrictions from the valves, and reduce the complexity of the control loop by using less number of components, hence achieving lower weight, smaller size, and faster actuation speed, comparing with the conventional pump-valve approaches. For instance, to drive six joints independently, using a conventional one-pump-multi-valve (OPMV) approach would require a total component weight of 1527 g (two gear pumps and twelve valves), while, in contrast, the total weight of the DPD approach is only 639.2 g (six peristaltic pumps), or 2.39 times weight reduction. Two systems were set to test the actuation speed of actuators as shown in Figure 5E. In addition to the drastic weight difference, these two systems have also shown different flow rates in drawing water. The motion rate of the OPMV system is 6.14°/s, while the proposed DPD system reached 7.86°/s with 28% increase.

**FIGURE 6:**
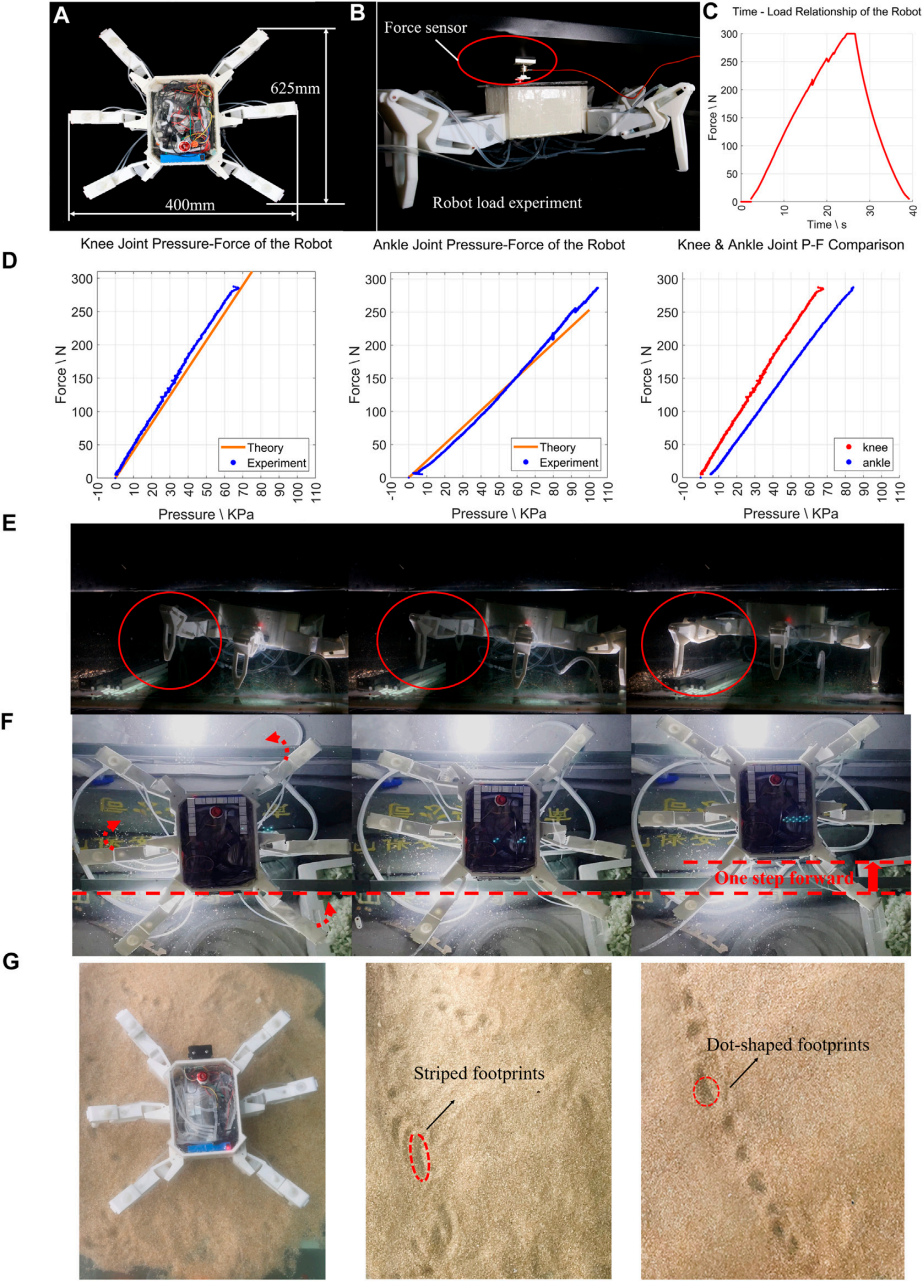
Tests on underwater crawling robot. **(A)** The underwater robot system size; **(B)** robot-loading experiment device; **(C)** robot load test results. **(D)** The results of all the knee joints and all the ankle joints tested individually. **(E)** The real scene of the robot moving before going down the water. **(F)** The process of robot crossing obstacles underwater (full motion clip in Supplementary Video). **(G)** Footprints of the robot walking in sandy water floor, showing different gaits.

Finally, the prototype of robotic system, with DPD control, is shown in Figure 6A. It consisted of six H^2^SA leg and one untethered body containing battery, pumps, and control system. The total weight was 5.75 kg. The dimensions were 625 mm in length and 400 mm in width.

The waterproofing of the U^2^C robot was significantly simpler than conventional motor-driven robots. The most crucial factor to be considered by conventional robots, the motion joints, were naturally water-sealed already with their enclosed body chambers, therefore requiring no further treatment to work underwater. Therefore, the remaining requirement was to treat the body compartment to be waterproof for the actuation and control components contained. In this work, the body compartment was fabricated using 3D-printed PLA. To waterproof it, a thin layer of coating resin (SMOOTH-ON XTC-3D) was applied to the entire body inside and outside, allowing sufficient curing time. The water pipes connecting to the joints were inserted from the ports at the bottom of the body compartment, and a layer of epoxy resin potting glue (7,026) was applied locally to fill the gap between the water pipe and the printed body part. Finally, a cap (3D-printed PLA) was fitted on the top of the body compartment with an O-ring made of silicone rubber to seal the gap in between (shown in Figure 5F). The entire set of hardware systems were enclosed into the body compartment, including six peristaltic pumps, a STM32 microcontroller, an emergency switch installed on the back of the robot, 12V lithium battery, and hydraulic pipes for connecting the actuation systems (shown in Figures 5D,G), making the system fully self-contained and untethered underwater.

### Tests on the System

A series of experiments were conducted to verify the performance of the U^2^C robot with the proposed H^2^SA leg design and DPD actuation. Two main aspects were validated: the payload, and the walking gaits. For the payload aspect, we aim to verify that by reinforcing the soft actuators with rigid components (proposed in the H^2^SA leg design), we could achieve significantly improved payload capability to be self-contained with extra load carrying margins; while for the gait aspect, we aim to verify that the H^2^SA leg and DPD actuation could overcome the large nonlinearities and excess compliance of soft robot limbs, and achieve gait patterns more similar to conventional motor-driven underwater robots, with linear or quasi-linear leg motions.

To test the load capacity of the robot, the system load test was conducted as set in Figure 6B in the air to avoid influence of buoyancy. Place the robot on the ground while placing a fixed plane above the robot. A force sensor is placed between the robot’s body and the plane, which can measure the upward push of the robot and thus the load of the robot. In the test, we added hydraulic pressure to all the knee joints and ankle joints at the same time. The soft joint generated forces to the ground by the robotic legs, which exerted an opposite force to the plane through the body. We have obtained the experimental results shown in Figure 6C. Analyzing the results of all the knee joints and all the ankle joints tested individually, they are highly coincident with the theoretical results shown in Figure 6D. From the experimental results of the robot system load, we have obtained the maximum load value of the robot as 300N, 5.22 times of the robot’s own weight of 57.5 N. This verified our design aim that with the proposed H^2^SA approach, the robot with soft actuators could not only support its own untethered weight, but also leave over 5 times load-carrying margin, remarkable especially for soft robots.

Finally, the locomotion of the U^2^C robot was shown with a series of gait demonstrations (clips included in the Supplementary Video). The alternate sequence could be achieved with the following eight steps, as shown in Figure 5B: 1) first evacuate all the air in the soft actuator; 2) evacuate water from the soft actuator a and inject water to the soft actuator d; 3) inject water to the soft actuators b and c and evacuate water from the soft actuator a, inject water to soft actuator d; 4) inject water into the soft actuators b and c; 5) evacuate water from the soft actuators e and f; 6) evacuate water from the soft actuators d, inject water into the soft actuators a; 7) inject water into the soft actuators e and f, evacuate water from the soft actuators d and inject water into the soft actuators a; 8) inject water into the soft actuators e and f. The above process completes a cycle of straight or turning gait. Setting the number of cycles can make the robot move in a preset range. With this sequence, the robot was put into a pool with some sediments for robot walking and obstacle crossing tests shown as Figures 6E,F. Figure 6E shows the leg movement in an underwater environment. To demonstrate the ability of the robot to jump over obstacles, the robot has the compliance of its legs when stepping on obstacles. Instead, it adapted to the complex environment through the adaptability of the soft robot, overcoming obstacles while keeping the gait of the entire leg unchanged (Figure 6F), as well as performing multiple different gait patterns over sandy surfaces (Figure 6G). This demonstrates the characteristics of the proposed H^2^SA leg design: despite using soft actuators in its joints, the leg could both achieve 1) quasi-linear leg motion; 2) large payload several times its own weight; and 3) passive compliance sufficient for overcoming obstacles. This unique combination would potentially lead to lightweight and safe U^2^C robots with precise gait control and large payload capabilities.

## Conclusion and Future Work

In this work, we expanded the soft-robotic approach well proven in underwater manipulator and gripper designs, to explore the feasibility and efficacy in using soft robotic actuators to designing underwater crawling robots. To this end, a series of technical hurdles have been overcome by the proposed novel designs and control approaches, toward lightweight, compact, multi-gait crawling robot platform design, and high-load capabilities. By using rigid structural components to strategically reinforce the otherwise omni-directionally flexible soft actuators, their loading capability and actuation precision could be drastically increased. Furthermore, by augmenting soft actuators into rigid leg links strategically, we could achieve quasi-linear motions despite the inherent highly nonlinear soft actuator characteristics with multiple times force-to-self-weight ratio, while maintaining excellent passive impact compliance of soft actuators. Completed with the proposed DPD actuation approach, we designed an untethered, lightweight crawling robot prototype with 300 N payloads, or a 5:1 payload-to-weight ratio and multi-gait capability.

Analytical models for joint, leg, and robot design were derived as references for robot design and verified against experimental data in the series of tests conducted on dedicated experimental platforms using the robot joints, legs, and over the fabricated robot prototype. To further evaluate the performance of robotic system, a series of pool tests were conducted, showing the crawling robot’s ability to cross the obstacles by passive compliance. The methodology could be concluded as follows: 1) The proposed method of modular combination of soft actuator joints was effective for underwater crawling robots. The proposed H^2^SA leg could achieve 4.5 kg payload with a self-weight of 286.6 g, while also achieving dexterous motions of up to 60 degrees of bending in each joint. Based on the single-leg design, the underwater crawling robot can be customized through the modularization of the soft robot joints, achieving various postures with identical modular actuators. 2) The proposed coordinated motion of the H^2^SA knee and ankle joints could achieve outstanding trajectory along the vertical axis during walking gaits. In particular, during the entire range of leg descending and ascending, the maximum lateral displacement of the leg tip was 16.8% of the vertical lifting range. The resulting leg motion could reduce the amount of disturbance to the ground surface while using three instead of four actuators in each leg design could effectively reduce the actuation system by 25%. 3) The proposed untethered robot system integrates the pump valve system into the robot body. The total weight of the robot including the actuation system is 5.75 kg. Through experiments, it is measured that the maximum payload of the system is 30 kg, and the weight-to-weight ratio is 5.22 times. With the untethered robot already fully self-contained, the resulting payload could be fully devoted to load carrying, providing the proposed crawling robot design a vast potential in underwater operation applications.

Future work will build on the demonstrated feasibility of using soft robotic actuators in underwater platform design, and explore further design variations on both the joints and legs. Owing to the limited scope of this article, further explorations regarding the actuation and control for the proposed robot were preliminary; they will remain to be refined together with other refinements on the robot design. Finally, the gait analysis and design of the robot could be explored further. With the demonstrated potential of the soft actuator legs, this design approach could potentially enable precise, lightweight, and passively safe crawling robots that could carry a high amount of payload to explore ocean floors for various benthic operations.

## Data Availability

The original contributions presented in the study are included in the article/Supplementary Material; further inquiries can be directed to the corresponding authors.
